# Comparison of clinical burden between patients with erosive hand osteoarthritis and inflammatory arthritis in symptomatic community-dwelling adults: the Keele clinical assessment studies

**DOI:** 10.1093/rheumatology/ket267

**Published:** 2013-09-17

**Authors:** Wing-Yee Kwok, Margreet Kloppenburg, Michelle Marshall, Elaine Nicholls, Frits R. Rosendaal, Danielle A. van der Windt, George Peat

**Affiliations:** ^1^Department of Rheumatology, Leiden University Medical Center, Leiden, The Netherlands, ^2^Arthritis Research UK Primary Care Centre, Keele University, Keele, UK and ^3^Department of Clinical Epidemiology, Leiden University Medical Center, Leiden, The Netherlands.

**Keywords:** erosions, hand osteoarthritis, inflammatory arthritis, pain, function

## Abstract

**Objective.** To investigate in the general population the clinical impact of erosive OA in interphalangeal joints (IPJs) compared with symptomatic radiographic hand OA and inflammatory arthritis.

**Methods.** Standardized assessments with hand radiographs were performed in participants of two population-based cohorts in North Staffordshire with hand symptoms lasting ≥1 day in the past month. Erosive OA was defined as the presence of an eroded or remodelled phase in ≥1 IPJ using the Verbruggen–Veys method. Radiographic hand OA was defined as the presence of ≥1 IPJ/first carpometacarpal joint with a Kellgren–Lawrence score of ≥2. Diagnoses of inflammatory arthritis were based on medical records. Hand pain and disability were assessed with the Australian/Canadian Hand Osteoarthritis Index (AUSCAN). Linear regression analyses were used to compare clinical determinants between groups and calculate mean differences with 95% CIs, adjusted for age and sex.

**Results.** Of 1076 participants with hand symptoms [60% women, mean age 64.8 years (s.d. 8.3 years)]; 80 persons (7.4%) had erosive OA. The population prevalence of erosive OA in ≥1 IPJ was 2.4% (95% CI 1.8, 3.0). Persons with erosive OA reported more pain and disability than persons with symptomatic radiographic hand OA [adjusted mean difference 1.3 (95% CI 0.3, 2.3) and 2.3 (95% CI 0.4, 4.2), respectively]. Individuals with inflammatory arthritis (*n* = 44) reported more pain and disability than those with erosive OA [adjusted mean difference 1.7 (95% CI 0.05, 3.4) and 6.3 (95% CI 2.8, 9.9), respectively].

**Conclusion.** While erosive OA has a greater impact than symptomatic radiographic hand OA in the general population, it is not as severe in terms of hand pain and disability as inflammatory RA.

## Introduction

Erosive OA of the hand is thought to be a subset of hand OA [1] and was first described by Peter *et al.* in 1966 [2]. The clinical features in erosive OA can appear as pain, swelling, redness, warmth and limited function of the interphalangeal joints (IPJs), which can be absent in non-erosive OA [3]. However, it is only recently that research into the occurrence of erosive OA in large-scale epidemiological studies has become possible with the development and validation of standardized methods for scoring cardinal features of IPJs, central erosions and collapse of the subchondral bone plate on radiographs [4–6].

The Rotterdam cohort was one of the first studies to provide a population prevalence of erosive OA in the IPJs of 2.8% of adults age ≥55 years in the general population, equivalent to 1 in 10 people with symptomatic hand OA [7]. Shortly after this, the Framingham Study showed age-standardized prevalence estimates for erosive OA of 9.9% in women and 3.3% in men [8]. These, and other previous studies in clinical populations, have consistently found more severe symptoms and functional limitations among those with erosive OA than those with non-erosive OA [7–10], raising the concern that erosive OA may carry the same burden as seen in inflammatory arthritis. This concern was mainly raised by studies performed in rheumatology practices in secondary and tertiary care comparing patients with hand OA with patients with RA [11, 12]. In rheumatology practices, the proportion of patients with erosive OA is relatively high. In these studies the clinical burden was similar between patients with hand OA and RA. However, a study comparing patient groups referred to a rheumatology outpatient clinic may lead to selection bias, since the high clinical burden in itself can be a reason for referral.

The aims of this study were to confirm the prevalence of erosive OA in a general population sample in the UK, to explore the impact of erosive OA on clinical outcomes further and to investigate the clinical impact of erosive OA compared with inflammatory arthritis arising from a population-based UK cohort with hand symptoms.

## Methods

### Population and study design

Data were collected from the Clinical Assessment Study of the Hand (CAS-HA) and Knee (CAS-K), both prospective, population-based, observational cohort studies in North Staffordshire, UK. The protocols of these studies are described elsewhere in detail [13, 14]. In short, all adults age ≥50 years registered with two general practices were invited to participate in a two-stage postal survey. If they indicated that they had experienced hand pain or hand problems within ≤12 months on the first postal questionnaire they were invited to the research clinic. Those who attended the research clinic were included in the CAS-HA study (*n* = 623) [13]. CAS-K participants (*n* = 819) were recruited from a further three different general practices using recruitment methods identical to CAS-HA, except that participants were invited for a clinical assessment in the CAS-K study if they reported knee pain (rather than hand pain or hand problems) within the last year [14]. Ethical approval for the CAS-HA and CAS-K studies was obtained from the North Staffordshire Local Research Ethics Committee and all participants gave written consent for those studies. No patient consent or ethical approval was obtained for this study, as the data are based on CAS-K and CAS-HA studies. Only CAS-HA or CAS-K participants who indicated that they experienced hand symptoms (pain, aching, stiffness) ≥1 day during the past month are included in this article. This criterion was selected in order enable comparison of prevalences with the Rotterdam Study [7], where patients with hand pain during the past month were selected (instead of using the selection of pain during the past year).

### OA definitions

Radiographic hand OA was defined as a Kellgren–Lawrence (KL) score of ≥2 in at least one IPJ or the first carpometacarpal joint (CMCJ). Symptomatic radiographic hand OA was defined as having hand symptoms (pain, aching or stiffness ≥1 day during last month) and radiographic OA. Erosive OA is defined as having one or more E or R phase according to Verbruggen–Veys in the distal IPJ (DIPJ), proximal IPJ (PIPJ) or first IPJ.

### Radiographic assessment and scoring

Plain radiographs were completed of each hand in a posteroanterior (PA) view [13]. Distal, proximal and thumb IPJ (DIP, PIP and first IPJ) and first CMCJ were scored by two trained assessors (M.M. scored, *n* = 521; June Hand scored, *n* = 555) blinded for clinical data. Joints were scored for the presence and severity of OA with the KL score (range 0–4) [15]. Both observers re-scored 50 pairs to calculate inter- and intra-observer reliability. Inter-observer reliability for the presence of hand OA was moderate (κ = 0.5, percentage agreement 90%). The intra-observer reliability for the presence of hand OA was excellent (κ = 0.92 and 0.85, PA 98% and 98% for readers 1 and 2, respectively).

Erosions were scored by the Verbruggen–Veys scoring method [5] and defined as having eroded (E phase) or remodelled, irregular, sclerotic subchondral plates (R phase) in DIPJs, PIPJs and first IPJs. The Verbruggen–Veys scoring does not include first IPJs; however, the same rules for DIPJs and PIPJs were applied to this joint, again permitting direct comparison with the Rotterdam Study [7]. Erosions were scored by a single reader (W.K.), blinded for clinical data. The intra-observer reliability for erosions as a dichotomous variable in the Verbruggen–Veys scoring method was excellent (κ = 0.94) [16].

### Sample selection for scoring erosive disease in hand radiographs

The majority of hand radiographs were scored for erosions; exceptions were those radiographs that had no or very few OA features. The assumption was that erosions are not present in subjects with (almost) normal radiographs. To determine the selection for scoring erosions, KL scores in the DIPJs, PIPJs, first IPJs and first CMCJs were summed to form an overall score (KLsum) for every participant. The population was divided in subgroups by the summation scores (range 0–72). All radiographs in subgroups with KLsum ≥3 were scored. Random samples of at least 10% of subgroups with KLsum <3 were screened for erosions.

### Diagnosis of systemic inflammatory arthritis

Three sources of information were used to identify potential cases of diagnosed systemic inflammatory arthritis—specifically RA, seronegative RA, PsA and scleroderma: retrospective local rheumatology hospital medical records, retrospective general practitioner medical records and the consultant radiologist’s clinical reports on participant’s study radiographs. All searches were conducted by a researcher abstracting information using a standard form and blinded to the study clinical assessments and, in the cases of the medical records reviews, the study radiographs. The abstracted information on potential cases was reviewed by members of the research team, including a consultant rheumatologist, to determine which diagnosis was made. These persons were used in the analyses of the comparison of clinical burden between erosive OA and inflammatory arthritis and were therefore excluded in the group used for erosive OA analyses only.

### Clinical outcomes

General characteristics of age and gender were recorded in postal surveys and height and weight were measured at the research clinics held at a local rheumatology outpatient department.

#### Hand pain and stiffness

The pain and stiffness subscales of the Australian/Canadian Hand Osteoarthritis Index (AUSCAN; range 0–20 and 0–4, respectively) were completed by all participants [17]. Self-reported pain was also assessed with the pain subscale of the Arthritis Impact Measurement Scales health status questionnaire (AIMS-2; range 0–10) [18]. Higher scores indicate more pain or stiffness. The presence of pain in the finger IPJs and the thumb was determined from hand drawings; participants shaded areas where they had experienced pain lasting ≥1 day during the past month.

#### Hand function and performance

Self-reported hand function was assessed with the function subscales of the AUSCAN (range 0–36) and AIMS-2 hand and finger function subscale (range 0–10). Higher scores represent a greater limitation in hand function. The maximum gross and pinch grip strength was assessed with the JAMAR dynomometer (Sammons Preston, Chicago, IL) and B&L pinch gauge (B&L Engineering, Tustin, CA), respectively. In addition, the Grip Ability Test (GAT) was performed in the CAS-HA participants [13]. The GAT consisted of three tasks (putting a flexigrip stocking over the non-dominant hand, putting a paperclip on an envelope, pouring water from a jug into a cup) that participants had to perform within 2–3 min [19, 20]. Scores are based on the time to complete the three tasks; higher scores correspond to poorer hand function. GAT scores <20 s are considered normal [19].

#### General health perceptions

General health perceptions were measured by the Short Form 12 (SF-12), a widely used generic health status questionnaire yielding summary component scores for physical health (PCS; 0–100) and mental health (MCS; 0–100), where lower scores represent poorer perceived health and a population average is 50 [21].

#### Aesthetics and impact of hand problems

The appearance of the hand was measured with the aesthetics subscale score of the Michigan Hand Outcomes Questionnaire (MHQ; range 0–100), which is composed of four questions for both hands [22]. The impact of hand symptoms was measured with the impact subscale of the AIMS-2 (range 0–10). Higher scores represent more satisfaction with aesthetics of the hand and a greater impact.

### Statistical analysis

The prevalence of erosive OA in the population with hand symptoms and in the symptomatic radiographic hand OA population was calculated by dividing the number of persons with erosive OA by the sample size. Associated 95% CIs were calculated based on a binomial distribution. The true population prevalence of symptomatic erosive OA was calculated using a combined approach of multiple imputation and weighted logistic regression, calculated for CAS-HA participants only [23]. Multiple imputation was used to estimate erosive OA prevalence in participants unable to attend the clinical assessment; weighted logistic regression was used to obtain prevalence rates adjusted for participant’s likelihood to return the initial survey questionnaire.

Linear regression analyses were used to investigate differences in clinical characteristics between participants with and without erosive OA and also those with erosive OA in comparison with those with inflammatory arthritis. The beta estimate is presented as the mean difference (with 95% CI) adjusted for age and gender. Data of participants with inflammatory arthritis were only used for the comparison of the clinical burden outcomes between participants with erosive OA and those with inflammatory arthritis of the hand and for estimates of overall population prevalence. The data were analysed using SPSS version 17 (SPSS Inc, Chicago, IL, USA) and STATA version 11.0 (Stata Corporation, College Station, TX, USA).

## Results

### Clinical characteristics and demographics

**Fig. 1:**
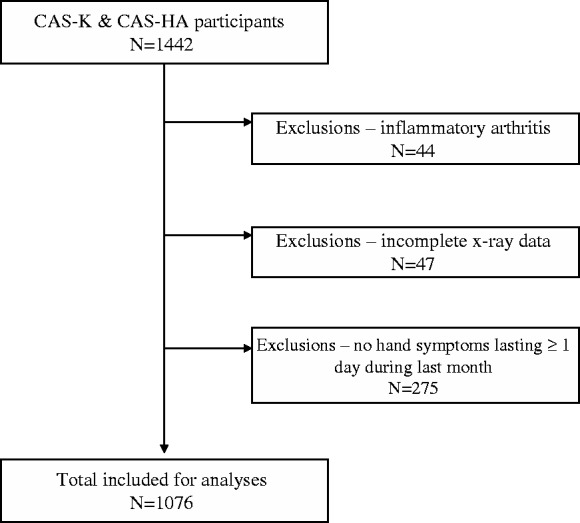
Flow chart of selection of CAS-K and CAS-HA participants for erosive OA analyses.

The cohorts yielded a combined sample of 1442 potentially eligible participants. Participants with incomplete radiographs (*n* = 47), without hand symptoms ≥1 day during the last month (*n* = 275) and those with inflammatory arthritis (*n* = 44) were excluded (Fig. 1), leaving a total of 1076 eligible participants [60% women, mean age 64.8 years (s.d. 8.3)]. The 44 persons with inflammatory arthritis were used in the analysis of clinical burden between erosive OA and inflammatory arthritis. Symptomatic radiographic hand OA was present in 74% of participants (Table 1).

**Table 1:** Baseline characteristics of 1076 persons in the population with hand symptoms lasting ≥1 day during the last month

Female, *n* (%)	650 (60)
Age, mean (s.d.), years	64.8 (8.3)
BMI, mean (s.d.), kg/m^2^	29.1 (5.1)
Pain in at least one IPJ, *n* (%)	527 (49)
Pain in left or right thumb, *n* (%)	605 (56)
Symptomatic radiographic hand OA^a^, *n* (%)	798 (74)
Erosive persons^b^ with IPJ erosions, *n* (%)	80 (7.4)

^a^Presence of Kellgren–Lawrence score ≥2 in at least one DIPJ, PIPJ or first IPJ.

^b^Having at least one eroded (E phase) or remodelled joint (R phase), according to the Verbruggen–Veys scoring method.

### Occurrence of erosive OA

Among the 80 persons with ≥1 erosive or remodelled joint in their DIPJ, PIPJ or first IPJ, a total of 216 erosive or remodelled joints were found (median 2, range 1–11), most commonly in the second DIPJs in both hands (34 joints in DIP2 left, 39 joints in DIP2 right). The second PIPJs (one joint in PIP2 left and right) were least commonly involved. Of the 216 joints, 34 joints (16%) were in the E phase; the remainder was classed as R phase. Twenty-three persons presented one or more E phase in their hands and 57 persons presented only R phases. Of the 23 persons, 76 erosive or remodelled joints were present, whereas 140 erosive or remodelled joints were present in the 57 persons with only R phase.

The true population prevalence estimate of erosive OA in the general population of adults age ≥50 years was 2.4% (95% CI 1.8, 3.0). This represented 7.4% (95% CI 5.9, 9.2) of the subpopulation with hand symptoms in this age range and 10.0% (95% CI 7.9, 12.1) of those with symptomatic radiographic hand OA. The prevalence of erosive OA in IPJs in the subpopulation with hand pain in the IPJs was 15.2% (95% CI 12.1, 18.2) and in the subgroup with symptomatic radiographic IPJ OA it was 23.3% (95% CI 18.8, 27.7). The prevalence of erosive OA was examined by gender and it was found that estimates for women were at least double those for men (Table 2).

**Table 2:** Prevalence of erosive OA in the total population age ≥50 years in those with hand symptoms and symptomatic radiographic hand OA, stratified for sex

Prevalence of EOA	All	Males	Females
Total population age ≥50 years	2.4 (1.8, 3.0)	0.9 (0.3, 1.4)	3.7 (2.7, 4.7)
Subpopulation with hand pain	7.4 (5.9, 9.2)	3.1 (1.6, 5.2)	10.3 (8.0, 12.6)
Subpopulation with hand pain in IPJs as well (*n* = 527)	15.2 (12.1, 18.2)	7.3 (4.0, 12.2)	19.2 (15.1, 23.3)
Subpopulation with symptomatic RHOA	10.0 (7.9, 12.1)	4.5 (2.4, 7.6)	13.2 (10.2, 16.1)
Subpopulation with symptomatic RHOA in IPJs as well (*n* = 344)	23.3 (18.8, 27.7)	13.8 (7.6, 22.5)	26.8 (21.3, 32.3)

Numbers are percentages (range 0–100%) with 95% CI in parentheses. Subpopulation with hand pain: having pain of the hands ≥1 day during the last month; subpopulation with symptomatic radiographic hand OA: meeting the criteria for hand symptoms and at least one joint in the DIPJs, PIPJs or first IPJs or the first CMCJ with a Kellgren–Lawrence score ≥2; IPJs: including DIP, PIP or first IPJ. EOA: erosive OA; RHOA: radiographic hand OA.

### Clinical burden of erosive OA in relation to symptomatic radiographic hand OA

Persons with erosive OA reported significantly more pain, stiffness and functional limitations than persons with symptomatic non-erosive radiographic hand OA on both AUSCAN and AIMS-2 questionnaires (Table 3). The power grip and pulp pinch strength tended to be lower in persons with erosive OA than those with symptomatic radiographic hand OA, after adjustment for age and sex, but not significantly different. In the performance of the GAT, no significant differences in time taken to complete the tasks were found between persons with erosive OA and persons with symptomatic radiographic hand OA.

**Table 3:** Demographic characteristics and clinical outcomes in the symptomatic radiographic hand OA subpopulation (*n* = 798)

Outcome	Persons with symptomatic RHOA (*n* = 718), mean (s.d.)	Persons with EOA (*n* = 80), mean (s.d.)	Adjusted mean difference^a^ (95% CI)
Female, *n* (%)	442 (62)	67 (84)	22.2 (13.4, 31.0)
Age, years	66.1 (8.1)	69.2 (7.8)	3.1 (1.3, 5.0)
BMI, kg/m^2^	29.3 (5.1)	28.7 (5.1)	−0.6 (−1.7, 0.6)
AUSCAN pain	6.6 (4.2)	8.0 (4.2)	1.3 (0.3, 2.3)
AUSCAN stiffness	1.1 (0.9)	1.5 (1.0)	0.3 (0.1, 0.6)
AUSCAN function	10.4 (8.1)	13.8 (8.0)	2.3 (0.4, 4.2)
AIMS-2 pain subscale	3.8 (2.3)	4.7 (2.6)	0.8 (0.3, 1.4)
AIMS-2 hand/finger function	2.2 (2.1)	3.1 (2.4)	0.8 (0.2, 1.3)
AIMS-2 impact subscale	2.2 (2.1)	2.6 (2.2)	0.5 (−0.05, 1.0)
Power grip, lbs	50.7 (25.6)	37.4 (18.9)	−3.0 (−7.1, 1.1)
Pulp pinch, lbs	10.3 (4.1)	8.4 (2.7)	−0.3 (−1.0, 0.4)
GAT	31.8 (12.9)	32.3 (9.8)	−0.7 (−4.7, 3.4)
SF-12 PCS	37.6 (11.8)	37.0 (11.3)	0.5 (−2.4, 3.4)
SF-12 MCS	50.4 (10.8)	53.0 (9.3)	2.9 (0.2, 5.5)
MHQ aesthetics subscale	72.2 (20.5)	52.2 (23.7)	−17.6 (−22.8, −12.5)

Values are means (s.d.) unless stated otherwise, shown with the mean differences in outcomes between persons with and without erosive OA. EOA is erosive hand OA in one or more IPJs (including DIPJ, PIPJ or first IPJ).

^a^Adjusted for age and sex (exception: crude mean differences for age and sex), 1 lb = 0.453 kg.

No statistically significant differences were seen in the AIMS-2 impact subscale and PCS between persons with erosive OA and those with symptomatic radiographic hand OA. Persons with erosive OA scored significantly better on the MCS but worse on the MHQ aesthetics subscale than persons with symptomatic radiographic hand OA (Table 3). The results mentioned above did not change when the analyses were also adjusted for BMI.

### Clinical burden in different stages of erosive OA

Within erosive OA, those with only R phases reported less stiffness and better hand and finger function as assessed by AIMS-2 than persons with at least one E phase on the radiographs; also, self-reported hand function scores assessed by AUSCAN were lower, however, this difference was not statistically significant. There was no difference between E and R phases in pain, AIMS-2 impact subscale, MCS and MHQ aesthetic subscale. Furthermore, those with only R phases had a better perception of their perceived physical health than those with one or more E phases on their radiographs [adjusted mean difference 5.8 (95% CI 0.2, 11.5); Table 4]. When adjusted for BMI, the results did not change.

**Table 4:** Demographic characteristics and outcome measures of general health and disease-specific questionnaires and performance tests in erosive persons (*n* = 80)

Outcome	Erosive, ≥1 E phase (*n* = 23; 76 affected joints)	Erosive, R phase only (*n* = 57; 140 affected joints)	Adjusted mean difference^a^ (95% CI)
AUSCAN pain	8.7 (4.6)	7.7 (4.0)	−1.0 (−3.0, 1.0)
AUSCAN stiffness	2.0 (0.9)	1.3 (1.0)	−0.7 (−1.2, −0.2)
AUSCAN function	15.5 (7.9)	13.1 (8.1)	−2.4 (−6.4, 1.5)
AIMS-2 pain subscale	5.3 (2.8)	4.4 (2.5)	−0.8 (−2.1, 0.5)
AIMS-2 hand/finger function	3.9 (2.7)	2.8 (2.2)	−1.1 (−2.2, −0.1)
AIMS-2 impact subscale	2.5 (2.3)	2.6 (2.2)	0.1 (−1.0, 1.3)
SF-12 PCS	33.2 (11.1)	38.7 (11.1)	5.8 (0.2, 11.5)
SF-12 MCS	53.1 (9.5)	52.9 (9.3)	−0.3 (−5.1, 4.6)
MHQ aesthetics subscale	48.1 (23.7)	54.3 (23.7)	5.4 (−6.6, 17.3)

Values are mean (s.d.) unless stated otherwise, stratified for the presence of erosive (E) or remodelled (R) phase with mean differences of outcome between E phase and R phase persons. E phase: eroded joint according to the Verbruggen–Veys scoring method; R phase: remodelled joint according to the Verbruggen–Veys scoring method.

^a^Adjusted for age and sex; PCS: physical component summary score; MCS: mental component summary score.

### Clinical burden of erosive OA in relation to inflammatory arthritis

A total of 44 cases of pre-existing systemic inflammatory arthritis were identified (39 RA, 4 PsA, 1 scleroderma), with a mean age of 66.2 years (s.d. 9.3 years) and a mean BMI of 28.4 kg/m^2^ (s.d. 5.2 kg/m^2^). Sixty-one per cent were women, which is significantly lower than in the erosive OA patient group [mean difference −24.7% (95% CI −41.3, −0.8)]. In 36 patients this diagnosis had been made by a rheumatologist. The remaining eight relied on a combination of general practitioner diagnosis and consultant radiologist report on the study radiographs.

Compared with cases with diagnosed inflammatory arthritis, persons with erosive OA had less hand pain, stiffness and functional limitation on both the AUSCAN and AIMS-2 subscales. Persons with erosive OA also had better perceptions of both their physical and mental health than persons with inflammatory arthritis. No difference was seen in the MHQ aesthetics subscale score between persons with erosive OA and those with inflammatory arthritis (Table 5). The results did not change when adjusted for BMI.

**Table 5:** Clinical outcomes for participants with erosive OA and those with inflammatory arthritis (*n* = 80 and *n* = 44)

Outcome	Persons with EOA (*n* = 80), mean (s.d.)	Persons with inflammatory arthritis (*n* = 44)^a^, mean (s.d.)	Mean difference^b^ (95% CI)
Female, *n* (%)	67 (84)	26 (61)	−24.7 (−41.3, −0.8)
Age, years	69.2 (7.8)	66.2 (9.3)	−3.0 (−6.1, 1.6)
BMI, kg/m^2^	28.7 (5.1)	28.4 (5.2)	−0.3 (−2.3, 1.6)
AUSCAN pain	8.0 (4.2)	10.2 (4.1)	1.7 (0.05, 3.4)
AUSCAN stiffness	1.5 (1.0)	2.0 (0.8)	0.4 (0.02, 0.8)
AUSCAN function	13.8 (8.0)	20.3 (9.4)	6.3 (2.8, 9.9)
AIMS-2 pain subscale	4.7 (2.6)	6.1 (1.9)	1.2 (0.2, 2.2)
AIMS-2 hand/finger function	3.1 (2.4)	4.8 (2.9)	1.6 (0.5, 2.6)
AIMS-2 impact subscale	2.6 (2.2)	4.5 (2.9)	1.7 (0.8, 2.8)
SF-12 PCS	37.0 (11.3)	28.4 (9.5)	−8.4 (−12.9, −3.9)
SF-12 MCS	53.0 (9.3)	46.0 (11.3)	−7.3 (−11.5, −3.0)
MHQ aesthetics subscale	52.2 (23.7)	52.7 (27.5)	−1.3 (−11.6, 9.0)

Values are mean (s.d.) unless stated otherwise. EOA: erosive hand OA in one or more IPJs (including DIPJ, PIPJ or first IPJ).

^a^One person of the inflammatory arthritis category was missing.

^b^Adjusted for age and sex (crude mean differences for age and sex).

## Discussion

This study makes several contributions to current knowledge on the occurrence and impact of erosive OA. First, we have confirmed with a high degree of consistency, previous estimates of the prevalence of erosive OA in the general population. Second, we showed that in a population-based study, symptomatic subjects with erosive OA report more pain, functional disability and aesthetic damage as assessed with hand OA–specific questionnaires than symptomatic subjects with non-erosive radiographic signs. In this population-based study, erosive OA does not appear to impact as strongly on pain and function as prevalent inflammatory arthritis identified from the same population.

The additional value of the present study concerns the detailed assessments of the hand (e.g. clinical examination, AUSCAN, AIMS-2 and SF-12) in contrast to the Rotterdam and Framingham studies. The use of hand OA–specific questionnaires in this study makes it possible to quantify pain, functional limitation and health status in erosive OA in a general population sample with hand symptoms in more detail than previous studies have allowed. In both the Rotterdam and Framingham studies, a question about having hand pain or symptoms on most days [8] or during the last month was asked [7], while the Rotterdam Study, in addition, used the hand-specific questions of the HAQ [24, 25] to describe the increased disability in persons with erosive OA compared with the general population [7]. However, the HAQ includes more domains of functionality and these hand-specific questions were not validated in patients with hand OA [24, 25]. In the present study, the quantification of pain and function could be made since both AUSCAN and AIMS-2 were used, showing the same direction of the outcomes. Another advantage of the present study is the additional information obtained from the clinical examination and the SF-12, which extends the knowledge regarding the impact of erosive OA in people with symptomatic hand OA.

The prevalence estimates in the present study are very similar to those found in the Rotterdam Study. In the Rotterdam Study, 2.8% of adults age ≥55 years in the general population were estimated to have symptomatic erosive OA (equivalent to 6.9% in those with hand symptoms and 10.2% in the subgroup with symptomatic radiographic hand OA [7]). In the present study in adults age ≥50 years the estimates are 2.4%, 7.4% and 10.0%, respectively. Recently Haugen *et al.* [8] reported apparently higher prevalence estimates of erosive OA (9.9% for women and 3.3% for men ages 40–84 years) using data from the Framingham Study. These estimates were based on erosions defined by the Osteoarthritis Research Society International (OARSI) atlas, while the Rotterdam and Keele studies used the Verbruggen–Veys scoring method. More importantly, the Framingham estimates were of erosive OA irrespective of symptoms.

Persons with erosive OA experience not only more pain, but also more functional limitation and impact than those with symptomatic radiographic hand OA, measured with AUSCAN and AIMS-2 questionnaires. Scores of the AUSCAN subscales in the present study were slightly lower than those reported for persons with erosive OA in secondary care [9]. Regardless of the study population, all these studies confirm that persons with erosive OA have a higher clinical burden than persons with symptomatic radiographic hand OA. Persons with erosive OA did not report poorer overall perceived physical health than persons with hand OA, as reflected by the PCS. This finding is in line with Bijsterbosch *et al.* [9], who reported no difference in health-related quality of life in persons with erosive OA compared with persons with non-erosive OA.

The clinical burden of erosive OA is lower than prevalent inflammatory arthritis in this population-based study. Individuals with inflammatory arthritis experienced a higher clinical burden than persons with erosive OA in terms of pain, functional limitation and physical health status. Recently Wittoek *et al.* [26] showed that patients with erosive OA visiting a rheumatology clinic have more funtional impairment and pain compared with patients with controlled inflammatory arthritis. An explanation for this contrary finding could be selection bias due to the different setting of the investigation (general population *vs* secondary care). Furthermore, the patients with inflammatory arthritis in the present study could have a higher disease activity (however, this was not measured since this was not the aim of the present study) than the patients in the Belgian study. During the development of the Score for the Assessment and Quantification of Chronic Rheumatoid Affections of the Hands (SACRAH) questionnaire, which is a score for assessment and quantification of chronic rheumatic affections of the hand, the scores concerning function, pain and stiffness were not significantly different between 69 OA and 103 RA patients [11]. The finding of a lower perceived physical health status in persons with inflammatory arthritis is in line with a population-based study in Spain reporting mean PCS scores from the SF-12 in persons with RA of 29.1 compared with 35.5 in persons with hand OA, after adjustment for age and sex [27]. The study of Slatkwosky *et al.* [12] showed that patients with RA and hand OA score worse on the SF-36 compared with the general population, but RA patients score worse than OA patients (SF-36 score of 59.1 for hand OA patients, 48.4 for RA patients and 81.6 for controls, respectively). However, in all three above mentioned studies, direct comparison with erosive OA was not investigated. The novelty of the present study is that health-related quality of life, pain and function scales of the AUSCAN and AIMS-2 in persons with erosive OA were directly compared with persons with inflammatory arthritis from the same source population.

Several limitations in the present study deserve mention. Although both cohorts (CAS-HA and CAS-K) gathered comparable data, they were assembled in subtly different ways: one based on knee symptoms, the other on the basis of hand symptoms in the past 12 months. Biased estimates from the knee cohort would be a concern, although the difference in the frequency of erosive OA between the two cohorts was not large (8.1% in CAS-HA *vs* 6.8% in CAS-K), which justifies their combination. The identification of cases of inflammatory arthritis was based predominantly on a pre-existing recorded diagnosis by a rheumatologist. In the absence of a thorough diagnostic screen for all inflammatory arthritis in the research clinics (which was beyond the scope of the present study), there could be the potential for some cases of inflammatory arthritis to have been missed due to incomplete records or early arthritis not yet diagnosed. Also, no specific information about swollen tender joints (such as disease activity scores like the DAS28) was available.

Furthermore, the number of persons with erosive OA, differentiation between E and R phases and persons with inflammatory arthritis were small and results may not be significant due to these small numbers. However, no earlier studies investigated these groups in detail with specific outcomes. These results need to be confirmed in future studies. In conclusion, erosive OA in the general population is an infrequent hand OA subset that occurs mostly in the DIPJs, with a predominance in females, and has consistent and substantial impact on pain and self-reported function, although appearing not as great as in persons with prevalent inflammatory arthritis.

**Rheumatology key messages**

- Erosive OA as a subset occurs infrequently in the general population, mostly in females and in the DIPJs.
- The clinical impact of erosive OA is less severe than inflammatory arthritis in a general population.
